# Multicenter assessment of quantitative sensory testing (QST) for the detection of neuropathic-like pain responses using the topical capsaicin model

**DOI:** 10.1080/24740527.2018.1525682

**Published:** 2018-10-23

**Authors:** Catherine E. Ferland, Chantal Villemure, Pierre-Emmanuel Michon, Wiebke Gandhi, My-Linh Ma, Florian Chouchou, Alexandre J. Parent, M. Catherine Bushnell, Gilles Lavigne, Pierre Rainville, Mark A. Ware, Philip L. Jackson, Petra Schweinhardt, Serge Marchand

**Affiliations:** a Quebec Pain Research Network, Université de Sherbrooke, Sherbrooke, QC, Canada; b Research Centre, Shriners Hospitals for Children-Canada, Montreal, QC, Canada; c Department of Anesthesia, Faculty of Medicine, McGill University, Montreal, QC, Canada; d Alan Edwards Pain Management Unit, McGill University Health Centre, Montreal, QC, Canada; e Division des Neurosciences cliniques et cognitives, centre de recherche CERVO, Université Laval, Quebec, QC, Canada; f Centre for Integrative Neuroscience and Neurodynamics, School of Psychology and Clinical Language Sciences, University of Reading, Reading, UK; g Département santé buccale, Faculté de médecine dentaire, Université de Montréal, Montreal, QC, Canada; h National Centre for Complementary and Integrative Health, NIH, Bethesda, MD, USA; i Centre de recherche de l’Institut universitaire de gériatrie de Montréal (CRIUGM), Montreal, QC, Canada; j Département de stomatologie, Faculté de médecine dentaire, Université de Montréal, Montreal, QC, Canada; k School of Psychology, Université Laval, Quebec, QC, Canada; l Department of Neurology and Neurosurgery, Faculty of Medicine, McGill University, Montreal, QC, Canada; m Centre de recherche du CHUS, Sherbrooke, QC, Canada; n Department of Surgery, Faculty of Medicine and Health Sciences, Université de Sherbrooke, Sherbrooke, QC, Québec, Canada

**Keywords:** neuropathic pain, quantitative sensory testing, multicenter study

## Abstract

**Background:**

The use of quantitative sensory testing (QST) in multicenter studies has been quite limited, due in part to lack of standardized procedures among centers.

**Aim:**

The aim of this study was to assess the application of the capsaicin pain model as a surrogate experimental human model of neuropathic pain in different centers and verify the variation in reports of QST measures across centers.

**Methods:**

A multicenter study conducted by the Quebec Pain Research Network in six laboratories allowed the evaluation of nine QST parameters in 60 healthy subjects treated with topical capsaicin to model unilateral pain and allodynia. The same measurements (without capsaicin) were taken in 20 patients with chronic neuropathic pain recruited from an independent pain clinic.

**Results:**

Results revealed that six parameters detected a significant difference between the capsaicin-treated and the control skin areas: (1) cold detection threshold (CDT) and (2) cold pain threshold (CPT) are lower on the capsaicin-treated side, indicating a decreased in cold sensitivity; (3) heat pain threshold (HPT) was lower on the capsaicin-treated side in healthy subjects, suggesting an increased heat pain sensitivity; (4) dynamic mechanical allodynia (DMA); (5) mechanical pain after two stimulations (MPS2); and (6) mechanical pain summation after ten stimulations (MPS10), are increased on the capsaicin-treated side, suggesting an increased in mechanical pain (*P* < 0.002). CDT, CPT and HPT showed comparable effects across all six centers, with CPT and HPT demonstrating the best sensitivity. Data from the patients showed significant difference between affected and unaffected body side but only with CDT.

**Conclusion:**

These results provide further support for the application of QST in multicenter studies examining normal and pathological pain responses.

## RÉSUMÉ

**Contexte**: L’utilisation de tests sensoriels quantitatifs (QST) dans les études multicentriques est limitée, en partie à cause de l’absence de procédures normalisées au sein des centres.

**But**: évaluer l’application du modèle de la douleur traitée par capsaïcine en tant que modèle expérimental humain de substitution pour la douleur neuropathique dans différents centres et vérifier les variations dans les mesures des tests sensoriels quantitatifs entre les centres.

**Méthodes**: Une étude multicentrique menée par le Réseau québécois de recherche sur la douleur dans six laboratoires a permis d’évaluer neuf paramètres de tests sensoriels quantitatifs chez 60 sujets en bonne santé traités par capsaïcine topique afin de modéliser la douleur unilatérale et l’allodynie. Les mêmes mesures (sans capsaïcine) ont été prises chez 20 patients atteints de douleur neuropathique chronique recrutés dans une clinique de la douleur indépendante.

**Résultats**: Les résultats ont révélé une différence significative entre la zone de peau traitée à la capsaïcine et la zone contrôle pour six paramètres : 1) le seuil de détection du froid (CTF) et 2) le seuil de perception de la douleur causée par le froid (CPT) étaient plus bas sur le côté traité par capsaïcine chez les sujets en bonne santé, ce qui indique une diminution de la sensibilité au froid, 3) Le seuil de perception de la douleur causée par la chaleur (HPT) était plus bas sur le côté traité par capsaïcine chez les sujets en bonne santé, ce qui suggère une augmentation de la sensibilité à la douleur causée par la chaleur; 4) l’allodynie mécanique dynamique (DMA), 5) la douleur mécanique après deux stimulations (MPS2) et 6) la somme de la douleur mécanique après 10 stimulations (MPS10) ont augmenté sur le côté traité à la capsaïne, ce qui suggère une augmentation de la douleur mécanique (p < 0,002). Le CDT, le CPT et le HPT ont démontré des effets comparables dans les six centres, le CPT et le HPT démontrant la meilleure sensibilité. Les données des patients ont révélé une différence significative entre le côté affecté et le non côté non affecté, mais seulement dans le cas du CDT.

**Conclusion**: Ces résultats soutiennent l’application de tests sensoriels quantitatifs dans les études multicentriques portant sur les réponses normales et pathologiques à la douleur.

## Introduction

Quantitative sensory testing (QST) has become a valuable psychophysical tool to assess pain in mechanistic studies in healthy volunteers,^1–3^ clinical studies for diagnostic purposes,^4,5^ and pharmacological studies for the efficacy of analgesic compounds.^6,7^ QST evaluates the integrity of pain pathways and mechanisms by identifying alteration or impairment of the somatosensory system,^8,9^ whereby results may be compared between individuals or areas within one individual (affected versus control body parts).

The use of QST in multicenter studies has been quite limited so far due in part to various methodological differences and lack of standardized procedures, measures, and equipment among centers.^1,10–12^ It is therefore essential to minimize these limiting factors in order to achieve high quality standards for comparability among centers. In 2006, the German Research Network on Neuropathic Pain published a comprehensive protocol to be reproduced among centers performing clinical trials. The protocol, consisting of seven tests examining 13 QST parameters, allowed for a complete somatosensory phenotyping of a subject within 1 h for two symmetrical body areas.^3^ This protocol was used to conduct a nationwide multicenter trial to establish age- and gender-matched absolute and relative QST reference values from 180 healthy subjects, to be used as control values in the assessment of patients with neuropathic pain.^3^

Neuropathic pain-like responses are frequently assessed in human experimental pain models. Topical capsaicin has been previously used for assessing therapeutic efficacy of analgesics by producing a unilateral effect on healthy subjects.^13–17^ Topical capsaicin application has proven to be useful to sensitize peripheral nociceptors specialized in detecting unpleasant noxious stimuli.^18^ This experimental model is reliable to assess differences in pain sensitivity among individuals.^17^ However, the use of QST employing the capsaicin pain model in multicenter studies has not been reported and would allow comparison of findings when used as a surrogate experimental human model of neuropathic pain.

The present study aimed to assess the application of the capsaicin pain model as a surrogate experimental human model of neuropathic pain in different centers and estimate the variation in reports of QST measures across centers. Additionally, the QST protocol was conducted in patients diagnosed with neuropathic pain. This comparison of findings in patients and healthy subjects was intended to consider the clinical relevance of the topical capsaicin model. This project was performed as an initiative of the Quebec Pain Research Network.

## Material and methods

The initiative of the Quebec Pain Research Network aiming at formulating QST guidelines began in 2009. In total, six independent laboratories with expertise in QST and one pain clinic took part in the study across four academic institutions (McGill University, Laval University, Montreal University, Sherbrooke University). Data collection occurred between 2011 and 2012. The study protocol was approved by the institutional ethics committees of all participating centers prior to the beginning of the study. Consent forms were signed by participants prior to the beginning of testing.

### Subjects

In six laboratories, ten pain-free subjects (five women, five men) were recruited and completed testing, for a total of 60 healthy subjects. Inclusion criteria consisted of healthy subjects aged 18 to 75 years old who had the ability to adequately understand and respond in English or French. Healthy subjects did not have any history of migraine, chronic pain, neurological disorders (such as traumatic brain injury, stroke, epilepsy, encephalopathy), degenerative disorders (Alzheimer’s, Parkinson’s), should not have taken any medication affecting vigilance, should not have worked in the health care system or with people suffering from pain, and should not have previously participated in a study in any of the participating laboratories.

Twenty patients diagnosed with chronic neuropathic pain were recruited from a tertiary pain clinic (Alan Edwards Pain Management Unit, Montreal, Quebec) and completed the protocol (seven women, 13 men). Inclusion criteria consisted of medically confirmed diagnosis of chronic neuropathic pain, aged 18 to 75 years old, with proficiency in English or French and capable of reading and giving written informed consent. Excluded from the study were patients for whom the etiology of chronic pain was not neuropathic and patients who had active cancer. Diagnoses of neuropathic pain included idiopathic neuropathic pain (peripheral asymmetric polyneuropathy), peripheral nerve damage or injury, syringomyelia, trigeminal neuralgia, ilioinguinal neuropathy, as well as cervical and thoracic radiculopathy. Among the 20 patients, 19 had unilateral pain symptoms and one patient diagnosed with cervical myelopathy had bilateral pain.

### Experimental procedures

#### Testing and control areas

For the healthy volunteers, using a pen with washable ink, a 3 × 3 cm area was marked on both forearms at the midpoint between the crease of the elbow and the wrist to ensure that testing across all measurements was performed on the same skin area. On the nondominant forearm of healthy participants, a thick layer of commercially available odor-free 0.075% capsaicin cream (CAPZASIN-HP distributed by Chattem) was applied to this 9 cm^2^ area to induce sensitization (i.e., the capsaicin-treated side). Capsaicin temporarily sensitizes the peripheral nociceptors and thus can transiently induce allodynia and hyperalgesia.^16^ After 20 min, the cream was removed and the skin was thoroughly washed with water. The untreated forearm was used as the control skin area (i.e., control side).

Patients were tested on a marked 3 × 3 cm skin area where pain was reported as being the most intense or interfered most with daily life (i.e., affected side). Capsaicin was not applied to patients. The skin area corresponding to the contralateral body location consisted of the control skin area (i.e., unaffected side).

### Quantitative sensory testing

The room temperature was kept between 20°C to 24°C during testing. A training session including each site experimenter responsible for administering the protocol was conducted by one of the QST experts trained in Germany (University of Mainz, University of Heidelberg). Several supervised practice sessions to master the administration of all tests, including standardization of all verbal instructions, took place prior to the beginning of the study. The protocol was modified from the skin and muscle sensitivity testing procedures described in 2006 by the German Neuropathic Pain Study Group.^3^ Tests were chosen and instructions were developed based on those used by the German group and modified by consensus among the investigators of the present study. Our modified QST protocol included ten parameters providing a comprehensive profile of somatosensory functions assessed with thermal and mechanical procedures. The material and equipment used were standardized across all study centers. Each test was performed on each side for every participant, always starting on the control or unaffected skin area, followed by testing on the capsaicin-treated or painful skin. For all parameters, the mean of three consecutive measurements on each side was calculated.

### Thermal stimulations

Thermal testing started with the cold detection threshold (CDT), followed by the warm detection threshold (WDT), the cold pain threshold (CPT), and, finally, the heat pain threshold (HPT). Thermal thresholds were measured using a 3 × 3 cm probe connected to the TSA 2001-II or a Pathway CHEPS apparatus (Medoc, Israel) and reported in degrees Celsius. All baseline temperatures (*t*°) were initially set to 32°C and programmed to gradually increase or decrease at a rate of 0.5 or 1°C/s, depending on the test performed. Once the baseline temperature was perceived as neutral by the participant (i.e., it was described as neither warm nor cold), the thermal testing commenced.

### Mechanical stimulations

Mechanical detection threshold (MDT) was assessed using standardized von Frey filaments (Touch-Test Sensory Evaluators) and data were reported in grams. Dynamic mechanical allodynia (DMA) for light touch was assessed with a standardized brush (Somedic SENSELab, Brush-05), and numerical pain intensity scores were reported directly by subjects with the use of a visual analog scale (scale of 0–200 with 0 indicating *no pain* and 200 indicating *intolerable pain*). On this scale, the score of 100 marks the limit between a strong sensation and a painful sensation.^19^ Mechanical pain summation with pinprick stimuli was assessed after two and ten stimulations (MPS2 and MPS10) with the Neuropen (Owen Mumford) with disposable Neurotips, and pain intensity was reported with the use of a visual analog scale (range 0–200). Vibration detection threshold (VDT) was assessed with the 64-Hz Rydel-Seiffer graded tuning fork applied to the skin. Pressure pain threshold (PPT) was assessed using the JTech Medical USA or Wagner manual algometer. Thresholds were reported in kilograms.

### Statistical analysis

Descriptive data were obtained for age, gender, tested side, and study center. For healthy subjects, differences between control (C) and capsaicin-treated (T) sides were assessed with the use of repeated measures analyses of variance (ANOVAs) with testing center and gender as between-subject co-variates. Prior to repeated measures ANOVAs, normality of variables was evaluated with the Kolmogorov-Smirnov test, and the following logarithmic transformation was applied for variables without normal distribution: for CDT ln [(max + 1) − CDT] and for MDT and DMA ln(variable + 1). Transformed data obtained a normal distribution. Effect sizes were calculated in the repeated measures ANOVAs models for tested sides. For QST variables where differences between control and capsaicin-treated sides were found to be significant in the repeated measures ANOVAs (effect of side), the proportion of subjects with similar response patterns for both tested sides (C and T) were examined.

Principal component analysis extracted from a correlation matrix was used to describe the variance structure of the tests with a significant effect of side. The analysis was performed on the differences between control and capsaicin-treated sides corrected for gender and testing center effects. If original data were nonnormally distributed, the difference was calculated between the two transformed variables.

Finally, repeated measures ANOVAs were also performed for data obtained in neuropathic pain patients. Those ANOVAs were composed of only two factors: (1) body side and (2) gender (note that all patients were recruited at a single site). *P* values less than 0.05 were considered statistically significant. Data are presented as mean ± standard deviation (SD) and deltas between control and capsaicin-treated sides. Statistical analysis was performed with SPSS software.

## Results

### Study participants

In all laboratories assessing healthy participants (identified as centers A to F), five female and five male participants were recruited. The mean ages (±SD) at each center were 21 ± 3.12, 22 ± 2.97, 23 ± 2.00, 23 ± 3.40, 22 ± 3.37, and 24 ± 4.49, for a global mean of 23.1 ± 5.1 years. The majority of participants reported having no pain at the time of testing, although three subjects reported presence of mild pain on the day of testing. No medical conditions were reported in any laboratory. One subject from center B had to stop the MPS10 assessment due to intolerable pain caused by the test. Results for this QST parameter are therefore reported for 59 subjects instead of 60.

The mean age for patients diagnosed with neuropathic pain (center G) was 54.0 ± 9.8 years. One patient with neuropathic pain had to stop the MPS2 and MPS10 measurements on the affected body side. Results for MPS2 and MPS10 are therefore reported for 19 patients instead of 20.

### Experimental procedures

A significant difference was observed in the mean time required to perform the entire protocol across the laboratories ranging from 59 to 73 min (F = 16.5, *P* < 0.001).

### QST measurements in healthy subjects

#### Thermal stimulations—Capsaicin effect

Significant differences were observed between the mean responses of the capsaicin-treated and control sides in three out of four thermal QST parameters (CDT, CPT, HPT; Table 1). The capsaicin-treated side showed reduced sensitivity to cool and cold pain (i.e., lower temperatures for CDT and CPT). In contrast, increased sensitivity was found to heat pain (lower HPT) and no significant effect was found for WDT.

**Table 1.** Repeated measure ANOVAs assessing effects of capsaicin-treated vs. control skin in healthy subjects for QST parameters with center and gender as confounders.

	CDT^a^	WDT	CPT	HPT	MDT^b^	DMA^b^	MPS2	MPS10	VDT	PPT
ANOVA factor (*P* values)										
Healthy subjects (*n*)	60	60	59^c^	60	59^c^	60	60	59^c^	60	60
Side (C vs. T)	<0.001	0.211	<0.001	<0.001	0.361	0.002	<0.001	<0.001	0.520	0.480
Gender	0.054	0.004	0.827	0.065	0.084	0.646	0.662	0.384	0.683	0.202
Center	0.130	0.102	0.411	0.217	0.200	0.003	<0.001	<0.001	<0.001	<0.001
Side × Gender	0.553	0.473	0.162	0.641	0.027	0.386	0.727	0.223	0.982	0.675
Side × Center	0.029	0.409	0.895	0.376	0.552	0.202	0.869	0.016	0.641	0.759
Effect size (C vs. T)	0.405	0.029	0.185	0.793	0.016	0.170	0.359	0.298	0.008	0.013

^a^ln[(max + 1) − CDT] transformation.

^b^ln(variable+1) transformation.

^c^One subject could not tolerate the test under that modality; see Materials and Method section.

ANOVA = analysis of variance; QST = quantitative sensory testing; CDT = cold detection threshold; WDT = warm detection threshold; CPT = cold pain threshold; HPT = heat pain threshold; MDT = mechanical detection threshold; DMA = dynamic mechanical allodynia; MPS2 = mechanical pain summation after two stimuli; MPS10 = mechanical pain summation after ten stimuli; VDT = vibration detection threshold; PPT = pressure pain threshold; C = control; T = capsaicin-treated.

#### Thermal stimulations—Testing center effect

**Figure 1.**
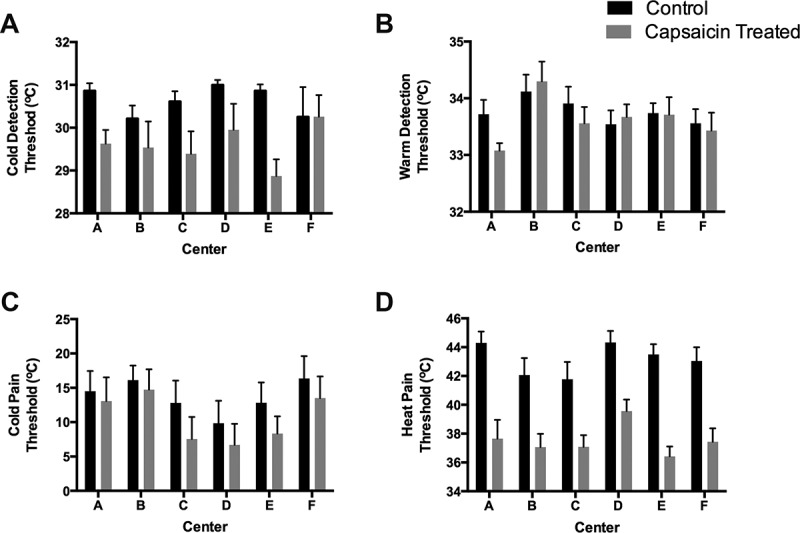
Thermal stimulation thresholds determined in six independent laboratories for capsaicin-treated and control sides of healthy subjects for (A) cold detection threshold; (B) warm detection threshold; (C) cold pain threshold; and (D) heat pain threshold. Data are presented as means ± standard errors of the mean of absolute temperature thresholds in degrees Celsius.

Across the six testing centers, mean values of the capsaicin-treated and control sides were comparable for CDT, WDT, CPT, HPT, and MDT (*P*s > 0.05, Table 1, Figure 1). The HPT was the most robust QST parameter measured with the lowest variability among all measures collected across all testing centers. When considering the difference between body sides tested (delta between control and capsaicin-treated), a significant difference was observed in ΔCDT among centers (*P* = 0.029), with one testing center reporting greater threshold values for the capsaicin-treated side. Other QST parameters did not show differences for the delta values across the centers (WDT, CPT, HPT).

#### Mechanical stimulations—Capsaicin effect

Three QST parameters were able to detect a significant difference between the capsaicin-treated and control body sides (Table 1). For MDT and DMA, the mean values were nonnormally distributed and a logarithmic [ln (variable+1)] transformation was performed to reduce skewness. Dynamic mechanical allodynia was significantly higher in the capsaicin-treated side compared to the control side (*P* = 0.002), indicating a greater pain sensitivity to brush-evoked pain on the capsaicin-treated side. Mechanical pain summation was illustrated by increased sensitivity in the capsaicin-treated skin after two (MPS2; *P* < 0.001) and ten (MPS10; *P* < 0.001) pinprick stimuli (Table 1). No differences were observed between sides for MDT, VDT, and PPT (all *P*s > 0.05).

#### Mechanical stimulations—Testing center effect

**Figure 2.**
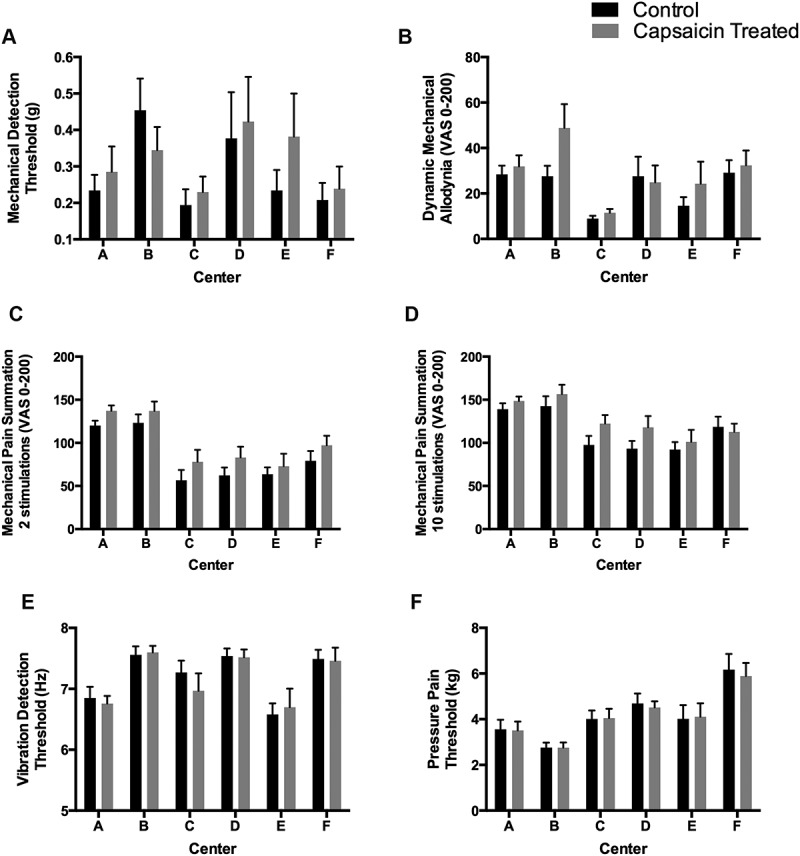
Mechanical stimulation thresholds determined in six independent laboratories (centers A to F) for capsaicin-treated and control sides of healthy subjects for (A) mechanical detection threshold; (B) dynamic mechanical allodynia; (C) mechanical pain summation (two stimulations); (D) mechanical pain summation (ten stimulations); (E) vibration detection threshold; and (F) pain pressure threshold. Data are presented as absolute threshold means ± standard errors of the mean.

Converse to thermal stimulation tests, the mechanical stimulation tests showed significant differences in the mean values between the six testing centers for DMA (*P* < 0.003), MPS (after both two and ten stimuli; *P* < 0.001), VDT (P < 0.001), and PPT (*P* < 0.001; Figure 2). When considering the difference between sides tested (delta between control and capsaicin-treated), a significant difference (*P* = 0.016) was observed among testing centers in the MPS10 deltas for testing sites A–F (Table 1).

Although no statistically significant differences were observed among all sites for MDT (*P* = 0.200), one center reported opposite results from all other centers with higher detection thresholds on the control body side (delta between control and capsaicin-treated of 0.02 at site B), when all other centers reported higher thresholds on the capsaicin-treated body side (Figure 2A). When testing for DMA, significant differences were observed in pain intensity reported by subjects between testing centers, with five centers reporting hyperalgesia on the capsaicin-treated skin, whereas one center reported hypoalgesia (site D; Figure 2B). Less variability among deltas was observed for the MPS2 parameter (Figure 2C) and MPS10 (Figure 2D), although again one center reported reverse results compared to the other testing centers.

#### Control and capsaicin-treated QST parameter response patterns

The results revealed differences in the parameter responses among the healthy subjects tested (Table 2). In fact, absolute threshold temperatures for CDT were lower on the capsaicin-treated side in 80% of subjects; that is, there was a reduced sensitivity to cool on the capsaicin-treated side compared to the control side, whereas 20% of healthy subjects had higher absolute threshold temperatures for CDT on the capsaicin-treated side, indicating an increased sensitivity to cool on the capsaicin-treated side. CPT values were reported to be lower in the majority of subjects (69.5%) on the capsaicin-treated side—that is, a decreased sensitivity to cold pain—although some subjects reported higher thresholds on the capsaicin-treated side (25.4%)—that is, an increased sensitivity to cold pain—and three subjects (5.1%) had similar values on both tested sides. HPT response patterns (control vs. capsaicin-treated) between both sides were similar for almost all subjects (96.7% had lower absolute temperature thresholds for HPT on the capsaicin-treated side, indicating an increased sensitivity to heat pain), with only two subjects out of 60 having higher HPT on the capsaicin-treated side; that is, a decreased sensitivity to heat pain. More variability in the response patterns was observed for the mechanical parameters, and higher sensitivity was reported on the capsaicin-treated body side for allodynia and pain summation (MPS).

**Table 2.** Proportion of healthy subjects and percentage of the total cohort with increased or decreased absolute threshold values in the capsaicin-treated body side in relation to the control body sides.^a^

Variable	C > T	C < T	C = T	*n*
CDT	48 (80.0)	12 (20.0)	0 (0.0)	60
CPT	41 (69.5)	15 (25.4)	3 (5.1)	59
HPT	58 (96.7)	2 (3.3)	0 (0.0)	60
DMA	16 (26.7)	36 (60.0)	8 (13.3)	60
MPS2	15 (25.0)	43 (71.7)	2 (3.3)	60
MPS10	16 (27.1)	39 (66.1)	4 (6.8)	59

^a^Data are presented as *n* (%).

C = control; T = capsaicin-treated; CDT = cold detection threshold; CPT = cold pain threshold; HPT = heat pain threshold; DMA = dynamic mechanical allodynia; MPS2 = mechanical pain summation after two stimuli; MPS10 = mechanical pain summation after ten stimuli.

We performed a principal component analysis of the six sensory tests that were significantly different between the control and capsaicin-treated sides in healthy subjects in order to investigate whether there was a pattern in the sensory profiles (Table 3). Among the original six variables, 33% of the total variance was explained by MPS2, MPS10, and DMA (component 1 of the principal component analysis). A strong association between these three tests was observed, suggesting that the three tests vary together. If MPS2 detects a strong difference between the control body side vs. the capsaicin-treated body side, MPS10 and DMA will likely differ as well between the two sides. CDT and CPT vary together (component 2), whereas HPT does not correlate with any of the original five sensory tests and by itself contributes 16.4% of total variance (component 3).

**Table 3.** Principal component analysis on the difference between the control vs. capsaicin-treated body sides (Delta (C − T)) of the six QST parameters that could detect a significant difference between C vs. T for healthy subjects (variables are corrected for site and gender effects).

Variable	Component 1	Component 2	Component 3
Delta (C − T) MPS2	0.787	0.182	0.304
Delta (C − T) MPS10	0.766	0.195	0.074
Delta (C − T) lnCDT	−0.256	0.810	0.159
Delta (C − T) CPT	0.523	−0.633	−0.060
Delta (C − T) HPT	−0.226	−0.248	0.926
Delta (C − T) lnDMA	0.636	0.298	−0.023
Proportion of variability of each component (%)	33.3	21.3	16.4

C = control; T = capsaicin-treated; QST = quantitative sensory testing; MPS2 = mechanical pain summation after two stimuli; MPS10 = mechanical pain summation after ten stimuli; CDT = cold detection threshold; HPT = heat pain threshold; DMA = dynamic mechanical allodynia.

### Chronic neuropathic pain patients

When assessing the difference between the affected and unaffected body sides of patients, the test to evaluate sensitivity to cold demonstrated reduced sensitivity (i.e., higher detection thresholds) on the affected body side with temperature values of 25 ± 8.3**°**C compared to 29 ± 2.0**°**C for the unaffected side (*P* = 0.022; Table 4). The CDT was the only parameter tested that was able to detect a significant difference between the affected and the unaffected body side (*P* = 0.022, effect size: 0.258).

**Table 4.** ANOVA and effect sizes comparing affected vs. non-affected sides and gender for different QST parameters in chronic neuropathic pain patients.

	CDT^a^	WDT	CPT	HPT	MDT^b^	DMA^b^	MPS2	MPS10	VDT	PPT
ANOVA factor										
Patients (*n*)	20	20	20	20	18	20	19	20	20	20
Side (affected vs. non-affected)	0.022	0.261	0.951	0.738	0.948	0.460	0.290	0.610	0.990	0.507
Gender	0.924	0.689	0.189	0.422	0.327	0.724	0.435	0.741	0.056	0.522
Side × Gender	0.513	0.763	0.424	0.756	0.897	0.331	0.693	0.318	0.730	0.478
Effect size	0.258	0.070	<0.001	0.006	<0.001	0.031	0.066	0.015	<0.001	0.025

^a^ln[(max + 1) − CDT] transformation.

^b^ln(variable+1) transformation.

ANOVA = analysis of variance; QST = quantitative sensory testing; CDT = cold detection threshold; WDT = warm detection threshold; CPT = cold pain threshold; HPT = heat pain threshold; MDT = mechanical detection threshold; DMA = dynamic mechanical allodynia; MPS2 = mechanical pain summation after two stimuli; MPS10 = mechanical pain summation after ten stimuli; VDT = vibration detection threshold; PPT = pressure pain threshold.

### Participants versus patients

**Figure 3.**
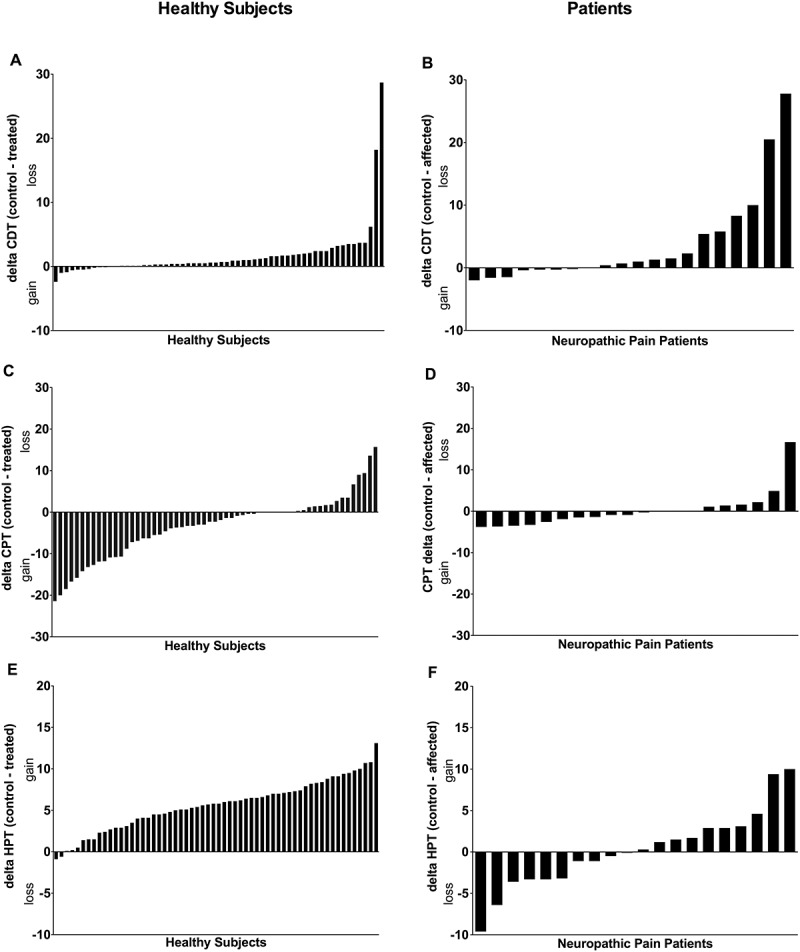
Interindividual patterns of thermal parameter results determined in six independent laboratories for control and capsaicin-treated skin in healthy subjects (*n* = 60) and control and affected skin for patients with chronic neuropathic pain (*n* = 20) for (A), (B) cold detection threshold, (C), (D) cold pain threshold, and (E), (F) heat pain threshold. Deltas of absolute values (control, capsaicin-treated skin) are presented for the healthy subjects and deltas (control, affected skin) are presented for patients with neuropathic pain. The direction of change (gain/loss of sensitivity) according to a positive or negative delta is indicated on the *Y*-axis.

**Figure 4.**
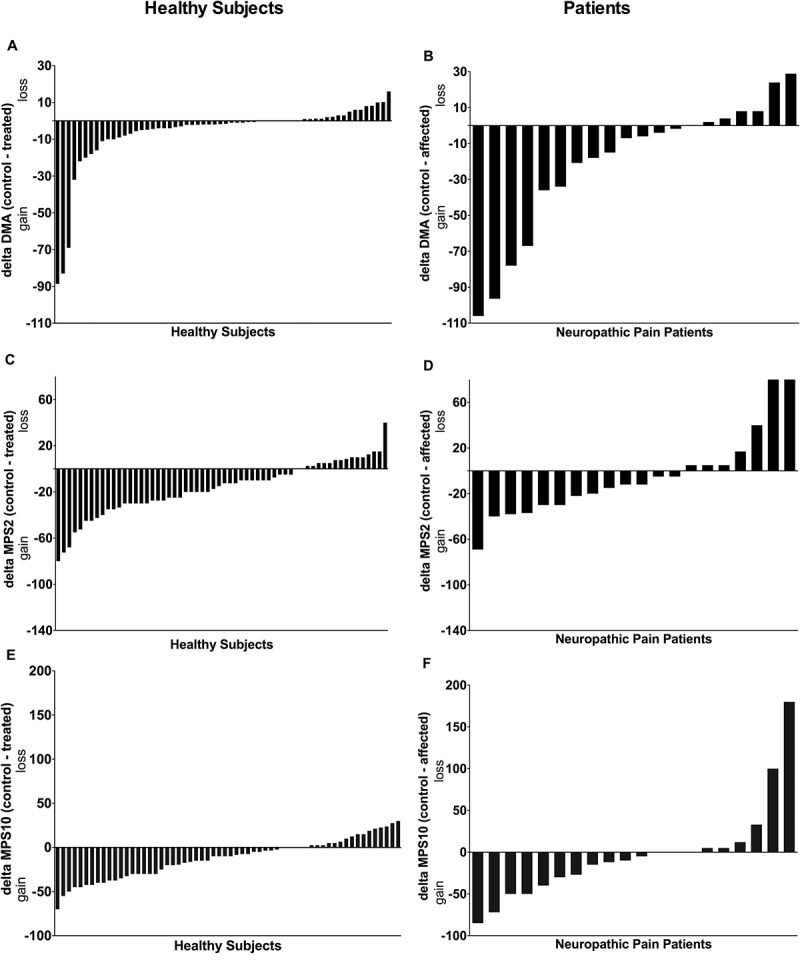
Interindividual patterns of mechanical parameter results determined in six independent laboratories for non-affected (control) and capsaicin-treated (treated) skin in healthy subjects (*n* = 60) and for control and affected skin in patients with chronic neuropathic pain (*n* = 20) for (A), (B) dynamic mechanical allodynia and mechanical pain summation after (C), (D) two stimuli and (E), (F) ten stimuli. Deltas of absolute values (control, capsaicin-treated skin) are presented for the healthy subjects and deltas (control, affected skin) are presented for patients with neuropathic pain. The direction of change (gain/loss of sensitivity) according to a positive or negative delta is indicated on the *Y*-axis.

Figures 3 and 4 illustrate the interindividual patterns by demonstrating parameter deltas (difference in the absolute threshold values) for the healthy subjects (control, capsaicin-treated skin) and deltas for the chronic neuropathic pain patients (control, affected skin). The relative proportion of healthy participants and neuropathic pain patients reporting the opposite pattern to the majority is very similar, with the exception of HPT and CPT.

## Discussion

Following a standard training session for the QST protocol, the capsaicin model was successfully implemented in six different participating centers. In our study, six out of nine QST measures detected a statistically significant difference between the capsaicin-treated and control body sides (CDT, CPT, HPT, DMA, MPS2, and MPS10). Among these, three were reproducible (CDT, CPT, and HPT) across the six testing centers. Thirty-three percent of the total variance of the difference between capsaicin-treated and control skin was explained by DMA and MPS, whereas HPT did not correlate with any of the other five sensory tests and uniquely explained 16.4% of the total variance. When considering the effect of testing center on capsaicin effects, only CPT and HPT were capable of detecting comparable differences between capsaicin-treated versus control sides across all testing sites. When assessing the same parameters in patients with chronic neuropathic pain, only CDT was significantly different between the two tested sides at the group level, with lower temperatures needed to be perceived at the affected side.

Abnormalities in somatosensory perception, including gain or loss of sensory function to thermal and/or mechanical stimuli, are frequent complaints of patients with peripheral neuropathies.^20–22^ In our study, the majority of healthy subjects reported hypoesthesia to cold stimulation on the capsaicin-treated skin in comparison to the control skin, which is a common symptom reported by patients with neuropathic pain^21,23^ and was also observed when testing with patients suffering from neuropathic pain in this study. However, when assessing pain sensitivity, we observed different patterns of sensory changes assessed by QST in healthy subjects and patients with neuropathic pain, although we did not perform a direct comparison between the QST measures of healthy subjects and patients with neuropathic pain due to a small sample size and differences in population source. A large proportion of our healthy subjects displayed an increased threshold (i.e., lower temperature) to cold-induced pain, suggesting cold hypoalgesia, whereas a lower cold pain threshold (i.e., higher temperature) has been reported in patients with unilateral neuropathy.^20,23^ Enax-Krumova and colleagues also reported a paradoxical capsaicin cold hypoalgesia.^24^ As they reported, we also expected signs of sensitization from capsaicin but also observed cold hypoalgesia, as in patients suffering from neuropathic pain. This concomitant phenomenon can be explained by the fact that the stimulation that was supposed to be on the primary zone was slightly offset and reached the secondary zone where sensitization has been reported in other studies.^25,26^ Several mechanisms could be responsible for this hyposensitivity, including the selective excitation of capsaicin-sensitive C fiber nociceptors likely leading to a selective spinal inhibition of mechanoreceptive nerve fibers.^26^

Nearly all healthy subjects showed increased pain sensitivity to heat stimuli, revealing the presence of thermal allodynia on their capsaicin-treated skin. Although heat hyperalgesia is found in patients with peripheral nerve injury, a larger proportion of patients show heat hypoalgesia.^23^ When applied to the skin, capsaicin triggers vesicular release of pro-nociceptive peptides in the periphery, leading to neurogenic inflammatory response.^27^ It is therefore not surprising that capsaicin induced an increased sensitivity to heat but not to cold, which is similar to inflammation.

Among the six sensory tests that differentiated capsaicin-treated from control skin, we observed a pattern of variation where certain tests seemed to be associated with each other and thus varied together. Cold sensitivity is mediated by A-delta and C fibers^8,28^ and when the tests yield measures corresponding to hypoesthesia, it is thought to be due to deafferentation.^8,28,29^ Capsaicin produces an initial algesic effect prior to the known reversible anti-nociceptive and anti-inflammatory actions.^30^ It seems that our results demonstrate the primary transient effects of topical capsaicin involving the vanilloid receptor subtype 1 activation on A-delta and C fibers. Although our healthy subjects did not have any nerve deafferentation, topical capsaicin produces temporary loss of cold sensitivity, yielding results for CDT and CPT similar to the sensory profile of patients with neuropathic pain with deafferentation.^17^ Similar to CPT, MPS measures the function of both A-delta and C fibers.^8,28^ The mechanism underlying temporal summation to pinprick stimuli (MPS10), as observed in the majority of subjects when comparing the capsaicin-treated body side to the control body side, is postulated to reflect central sensitization.^28,31^ Similarly, central sensitization is thought to be the neurophysiological process responsible for DMA.^32–36^ The fact that DMA and temporal summation to pinprick stimuli varied together suggests that the central phenomena of both measures are closely related. Similar to cold sensitivity, HPT tests the function of A-delta and C fibers.^8,28^ In contrast to the reduction in cold sensitivity, however, the majority of our subjects displayed heat hyperalgesia, indicating peripheral sensitization rather than deafferentation.^8,28,29^ This suggests that subpopulations of A-delta and C fibers were differently affected by capsaicin application. In support of this notion, differences in HPT between capsaicin and control skin did not co-vary with differences in CDT and CPT.

Capsaicin is a frequently used model to target pain therapies.^37^ However, whether capsaicin does reproduce a specific neuropathic-like sensory pattern is still under debate.^38^ The differential effects of capsaicin on the skin is dependent on the dose, duration, and frequency of application, possibly explaining how it can be both a therapeutic agent for pain as well as an experimental model of pain and in part how our QST results in healthy subjects did not entirely reproduce the sensory effects seen in Simone and Ochoa’s study.^39^ It has been used as an experimental model of neuropathic pain^17,37,40^ as well as a model of acute inflammation.^38,41,42^ Neuropathic pain is defined as pain caused by a lesion or disease of the central or peripheral nervous system.^22,43,44^ Therefore, it seems unlikely that capsaicin may accurately reproduce sensory abnormalities like those experienced by neuropathic pain patients, and its use as a surrogate model for neuropathic pain should be carefully considered. Capsaicin-induced irritation causes superficial dermal inflammation from an activation of vanilloid receptor subtype 1 receptors that is then conveyed through pain pathways, suggesting nociceptive pain rather than neuropathic pain.^44^ This nociceptive aspect of capsaicin may explain why we were unable to observe differences in some of the QST parameters, three of which were mechanical stimulations. If capsaicin only produces a topical nociceptive response, it then seems unsurprising that VDT and PPT were not discriminating between the capsaicin-treated and untreated body sides.

There was large variability observed in the interindividual results, suggesting that individuals react differently to topical capsaicin. In their study of healthy subjects, Lötsch et al. found that capsaicin induced a pain-like reaction similar to neuropathy in only 18% of their cohort (*n* = 110).^17^ This may explain the variability found in our study, specifically for CPT, where a significant proportion (25%) of the tested subjects reported lower pain sensitivity on their capsaicin-treated side. Therefore, capsaicin sensitivity prescreening test, as suggested by Lötsch et al., should occur prior to the actual experimental testing to ensure homogeneity in capsaicin sensitivity among study subjects.^17^

In line with the conclusions of the German Research Network on Neuropathic Pain,^8^ one of the strengths of this study was determining the gain or loss of somatosensory function by comparing an affected side (capsaicin-treated side in healthy subjects or painful side in patients) with its contralateral unaffected body side. This methodological approach contributes to eliminating some of the interindividual variance, providing a more accurate comparison between affected and unaffected body sides.

Certain limitations are worth noting. The lack of power may have caused missed discrimination between tested sides or may have caused missed differences among testing centers. Various studies have investigated the reliability and reproducibility of QST parameters in multicenter studies and have demonstrated their sensitivity to sensory abnormalities in patients experiencing neuropathic pain, confirming the validity of these tests.^21,23^ In our study, for some QST parameters measured, the interindividual variability was important enough that no differences were reported at the group level. Secondly, in the patient group, the lack of homogeneity in the syndrome underlying the neuropathic pain may have caused significant differences in the obtained results, because different neurological syndromes revealed different somatosensory profiles.^21,45^ A limitation of MPS is the choice of instrument used to produce pinprick pain. The shape of the Neuropen’s tip is both rounded and flat; a flat right-angled tip may be preferred to evoke pinprick hyperalgesia or sharp pain.^35^ However, it is believed that mechanical pinprick hyperalgesia is a response to stimulation of mainly A-delta fibers^46,47^ and this may not be the case with the Neuropen tip.^35^ Another potential limitation in our study was that no test for glucose tolerance was performed for the healthy participants. Diabetes mellitus and even impaired glucose tolerance are well-known causes of peripheral neuropathy.^48^

Differences between testing centers were observed for the majority of the assessed parameters, although a training session regarding the test procedures was conducted in each testing center. The experimental design followed recommendations by experts in the QST field, but the results still indicate some technical issues. Standardization seems to be more difficult when the experimenter is responsible for the stimulus (allodynia, summation, vibration, pain pressure), as previously suggested by Geber et al.^1^ Multicenter studies using QST in patients with pain and healthy subjects have shown poor reproducibility of thermal and mechanical thresholds.^12,49^ Furthermore, inconsistencies in acquired results could be attributed to the multi-examiner effect.^1^ They concluded that a strict standardized protocol and a rigorous training of examiners could overcome potential limitations of the multicenter approach.^1^ In our study, lab personnel were trained with a standardized protocol during a session, but no interobserver reliability was assessed. More emphasis on standardization of stimuli, instructions to the subjects, testing algorithms, and reference values (corrected for anatomical site, age, and gender) in healthy subjects and in patients are mandatory for quality multicenter studies.^50^

Most published studies report mean results for each group or treatment. However, there is an important variability between subjects. As shown in Figures 3 and 4, when plotting the delta between the control skin and the capsaicin-treated skin, there is substantial variability in the results for healthy subjects and patients. The subjects or patients with the highest and lowest responses most probably have different endogenous pain modulatory processes and may respond differently to a specific treatment.^51^ Taking into account this interindividual variability may guide the development of personalized approaches that would be better suited than approaches based on mean results.

In conclusion, capsaicin is a good experimental pain model that can be used for multicenter studies but with several limitations. Major precautions should be included in the experimental design. When using capsaicin, some QST thermal procedures provide better reproducibility across study centers.
